# COVID-19 stress and coping strategies among older adults: a systematic review of qualitative evidences

**DOI:** 10.1186/s40359-023-01382-1

**Published:** 2023-10-12

**Authors:** Amir Ahmadi, Hamid Allahverdipour, Sina Valiee, Fariba Pashazadeh, Nafiseh Ghassab-Abdollahi, Faranak Abdoli, Hossein Matlabi

**Affiliations:** 1 Department of Geriatric Health, Faculty of Health Sciences, Tabriz University of Medical Sciences, Tabriz, Iran; 2 Department of Health Education and Promotion, Tabriz University of Medical Sciences, Tabriz, Iran; 3 Research Center for Integrative Medicine in Aging, Aging Research Institute, Tabriz University of Medical Sciences, Tabriz, Iran; 4 Department of Occupational Therapy, School of Rehabilitation Sciences, University of Social Welfare and Rehabilitation Sciences, Tehran, Iran; 5 Clinical Care Research Center, Research Institute for Health Development, Kurdistan University of Medical Sciences, Sanandaj, Iran; 6 Research Center of Evidence-Based Medicine, Tabriz University of Medical Sciences, Tabriz, Iran; 7 Clinical Psychiatry Research Center, Tabriz University of Medical Sciences, Tabriz, Iran; 8 Student Research Committee, Tabriz University of Medical Sciences, Tabriz, Iran

**Keywords:** Older adults, COVID-19, Coping strategies, Systematic review

## Abstract

**Background:**

The COVID-19 pandemic has brought about far more stressful conditions for people worldwide. As a vulnerable group, older adults have suffered various psychological problems, such as stress, because of this pandemic and have applied various strategies to cope with the dire consequences. This study aimed to synthesize qualitative evidence regarding coping strategies for stressful situations among older adults throughout the COVID-19 pandemic.

**Methods:**

We searched electronic databases, including Scopus, Embase, PubMed, ProQuest, and the Cochrane Library, based on PRISMA standards. The protocol of this systematic review was registered on the PROSPERO (registration code: CRD42022364831). All relevant English-language articles published between 2019 and November 10, 2022, were searched. We reviewed the reference lists for all the included studies and key references. Two reviewers conducted screening, data extraction, and quality appraisal independently, with disagreements resolved by consensus with all team members. The Joanna Briggs Institute (JBI) checklist was used to assess the quality of studies. A thematic synthesis of the selected studies was conducted.

**Results:**

We included 13 studies in our review. Most studies were conducted in the early months of the COVID-19 pandemic. The stress caused by the COVID-19 pandemic was classified into six categories: health management challenges, stress caused by quarantine, economic challenges, media and bad news stress, virus threats, and challenges related to the use of communication technologies. The strategies used by older adults to cope with these challenges were categorized into five categories: protective strategies, avoidance strategies, maintaining social connections, meaning-based strategies, and fun strategies. This research showed that depending on the situation and conditions, older adults use various strategies to cope with COVID-19.

**Conclusion:**

Older adults experience much stress during the COVID-19 pandemic. In most cases, older adults can cope with these challenges with simple strategies from previous experiences and learnings. Older people require educational interventions in some cases, such as those involving communication skills. A better understanding of older adults coping strategies may enable policymakers to develop more effective policies and manage the problems of older adults in post-COVID situations.

**Supplementary Information:**

The online version contains supplementary material available at 10.1186/s40359-023-01382-1.

## Background

The COVID-19 pandemic has become a global public health crisis that has resulted in many challenges for the world. According to healthcare statistics, this virus has caused more than 645 million positive infections and more than 6.6 million deaths worldwide since the beginning of this epidemic [1, 2].

The psychological effects of high mortality and restrictive measures following the pandemic have changed the lifestyles of millions of people and created a unique combination of a living environment with unpredictable and stressful aspects, causing long-term, direct, and indirect effects on people’s physical and mental health [3]. The results of an extensive survey show that the stress level worldwide peaked during the COVID-19 crisis [4].

The COVID-19 pandemic is a potential source of increasing stress in older adults [5]. Coronavirus increases the risk of death in infected older adults. Additionally, older adults with underlying health problems, such as high blood pressure, heart problems, and diabetes, are at greater risk of contracting more severe types of COVID-19 and have been quarantined since the outbreak [6, 7]. Due to smaller social networks and less social support, the pandemic increased the risk of mental health problems among older adults more than other age groups [8].

A pandemic can cause various problems for the older adult, including much isolation, decreased contact with loved ones and the outside world, fewer opportunities to engage in favorite activities, and possibly elevated anxiety and fear of death. [9]. Financial stress, limited interactions with society, the loss of loved ones, exposure to negative media coverage, uncertainty about the future, and being discriminated against make older adults experience a high level of stress [9]. Other aspects of this pandemic, such as its unpredictability, have contributed to its stress and adversely impacted the mental health of older adults [10]. The reaction to an uncertain event depends on how a person evaluates and copes with the threat [11]. There are different ways that older people can deal with the stress of the COVID-19 pandemic [12].

Coping strategies are cognitive and behavioral efforts to explain, interpret, and modify a stressful situation to avoid or reduce suffering. *Coping* includes activities to reduce or endure psychological pressure [13]. A person’s strategies to cope with a stressful source such as COVID-19 are part of their vulnerability profile. Using an inappropriate strategy in facing stressful situations can increase stress while using correct coping strategies can have positive results [14, 15]. Stress assessment patterns and coping strategies among older adults may differ quantitatively (i.e., different means) as well as qualitatively (i.e., stress assessment patterns) [16]. A qualitative study with qualitative data is the best method for describing human experiences during pandemics [17].

Qualitative studies can effectively prevent secondary stressors during the COVID-19 crisis by highlighting people’s voices and clarifying their needs [18]. The systematic review of qualitative studies is a relatively new method in the systematic review of studies [19]. Systematic reviews of qualitative studies that analyze and categorize first-hand experiences of older adults living in the community during the COVID-19 crisis can provide a richer conceptual understanding of these experiences, and qualitative systematic reviews are capable of providing insights for healthcare professionals on topics and concerns which cannot be addressed solely through quantitative research. The impact of COVID-19 on the lives of older adults and their coping strategies may be better shown by studying the memories of seniors who engaged in numerous social and recreational opportunities outside of their homes before the quarantine. The results of this study can be used to plan for specialized services for older adults in the post-COVID era, as well as potential future pandemics. Therefore, the present study was to synthesize qualitative evidence regarding coping strategies for stressful situations among older adults throughout the COVID-19 pandemic.

## Methods

This study used the search strategy, screening, and data election based on PRISMA guide criteria [20]. The protocol of this systematic review was registered on the PROSPERO website to prevent duplicate work (registration code: CRD42022364831).

### Search strategy

The first author and an experienced academic librarian searched articles. Mesh keywords were used to search for related articles, and the keywords “coping strategy”, “coping strategies”, “older adults”, “elderly”, “aging”, “ageing”, “COVID-19”, “COVID-19 pandemic”, and “stress” were used. Scopus, Embase, Medline (via PubMed), ProQuest, The Cochrane Library, and Google Scholar were checked to find related English articles. This search was completed by checking the references of related articles.

### Study selection criteria

The study selection criteria included the following:

1. The study was conducted among older adults (60 years of age and older) living in the community.
2. The study was conducted with a qualitative or mixed methodology with an identifiable qualitative component.
3. The study looked into the coping strategies of older adults dealing with the stress caused by the COVID-19 pandemic.
4. The abstract and text of the study should be available in English.

**Fig. 1:**
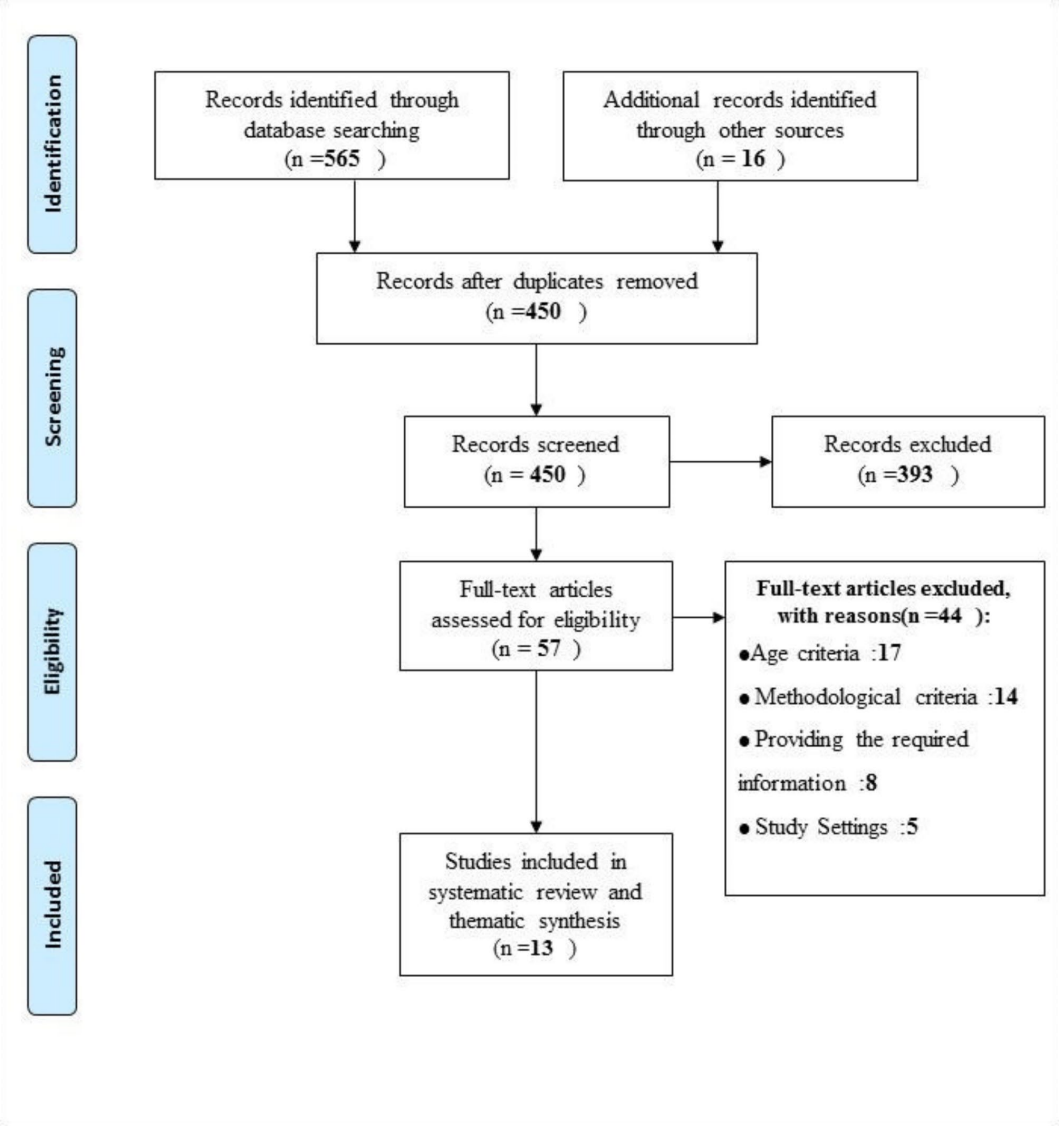
Steps of searching, screening, and selecting data based on the PRISMA chart

We use the PRISMA flow diagram to show our search results and the process of screening and selecting studies for inclusion (see Fig. 1). The identified articles were entered into EndNote 20. Duplicate articles were removed, and the remaining articles’ titles, abstracts, and full texts were screened to obtain the final list.

### Data extraction

Three researchers (A.A., H.M., and F.A.) independently performed the data extraction process using a pre-designed table based on the key features of the articles. The extracted information included the general characteristics of the study (name of the first author and year of publication, study location, sample size, age range of the study participants, data collection tool, and study date).

### Risk of bias

The quality of the selected articles was assessed using the Joanna Briggs Institute for Qualitative Research (JBI) Critical Appraisal Checklist. This tool helps researchers assess a study’s relevance, reliability, and methodological quality and ensures that the methodology, analysis, and interpretations are complementary. The JBI tool for evaluating qualitative studies has ten items [21]. Content points were scored as yes (2), no (0), and unclear (1). A decision was made to rank articles meeting 70–79% of checklist criteria as medium-high quality. Articles meeting 80–89% were identified as high quality, and those with a score of 90% and above were ranked as superior [22]. Two research team members (A.A. and H.M.) independently evaluated the methodological quality of the selected studies using the 10-item JBI checklist. Disagreements are resolved by consensus with all team members.

### Data synthesis

All study data were entered verbatim for qualitative synthesis in MAXQDA 2020. The process of combining themes was inductive. One of the researchers (A.A.) did preliminary coding using line-by-line coding to find related ideas and subtopics concerning the stress induced by the COVID-19 pandemic, and coping techniques for older adults were created based on this data. Then two researchers (H.M. and A.A.) independently coded the selected studies using this guide, and this work continued until no other sub-themes emerged. If there was a difference between the two researchers, the third researcher (H.A. or F.A.) discussed and judged it, and a consensus was formed.

## Findings

**Fig. 2:**
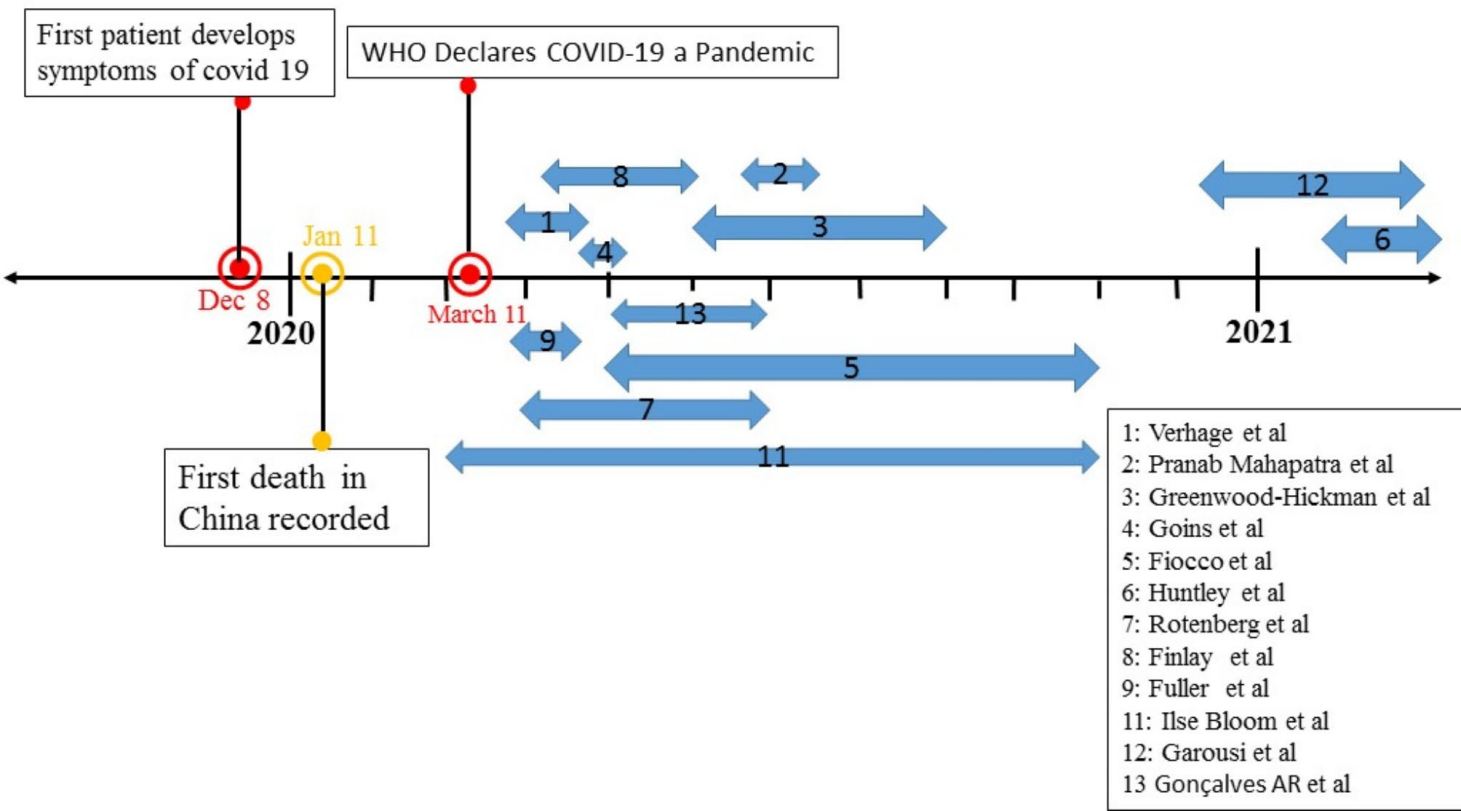
COVID-19 pandemic timeline and approximate time of data collection for selected studies

In the electronic database search, 565 studies were identified for screening. We also identified 16 other studies through a manual search in the references of the articles. The PRISMA flow chart is shown in Fig. 1. After removing duplicate articles and also checking the title and abstract of the articles, the full text of 57 studies was selected for further review, of which 44 studies did not meet the age criteria (n = 17), the methodological criteria (n = 14), the information requirements (n = 8), and the consistency of the study environment (n = 5). The remaining 13 articles that met all the inclusion criteria were included in the study [15, 18, 23–33]. Table 1 presents the characteristics of the selected articles. The largest number of articles (4 articles) were done in the USA [15, 23, 27, and 29], and other articles were conducted in Canada (two articles), Iran, England, India, Sweden, Uganda, and the Netherlands. Most studies used semi-structured telephone interviews to collect data [15, 18, 24, 26–30]. Except for one study, which did not specify when data were collected [31], all selected studies were conducted within months of the COVID-19 pandemic announcement (see Fig. 2).

**Table 1:** Summmary of selected articles

First Author And year of publication	Country	Mean and age range	Participant Profile	Type of study and Data collection tool(s)	Date of study
Miriam Verhage 2021	Netherlands	75.5 (54–95)	59 Older Adults 34 Females 25 Males	Qualitative study using semi-structured telephone interviews	Between 27 March and 20 April 2020
Pranab Mahapatra 2021	India	67 (60–85)	11 urban older adult couples living alone	reflective narrative approach using telephonic in-depth interviews	Last week of June to the middle of July 2020
Greenwood-Hickman 2021	USA	68 (60–77)	25 Older Adults 16 Females 8 Males 1 non-Binary	Qualitative study using semi-structured telephone interviews	Between June and August 2020
R. Turner Goins 2021	USA	72.4 (65–92)	43 Older Adults 24 Females 19 Males	Qualitative study using semi-structured in-depth interviews remotely	Between April 25, 2020 and May 7, 2020
Alexandra J. Fiocco 2021	Canada	72.2 (65–81)	22 older adults 13 Females 9 males	Qualitative research using semi-structured one-on-one interviews telephone or virtual interview using Zoom	Between May and October 2020
Richard Huntley 2022	Sweden	75.5 (71–82)	8 older adults 4 Females 4 males	Qualitative, explorative study using diaries and telephone interviews	Between February and March 2021
Shlomit Rotenberg 2021	Canada	71 (63–83)	16 Older Adults 12 Females 4 Males	Qualitative descriptive study using semi-structured interviews by phone	April–June, 2020
Jessica M. Finlay 2021	USA	67.3	5180 Older Adults 3302 Females 1870 Males	Qualitative content analysis using online questionnaire	From April 2 to May 31, 2020
Heather R. Fuller 2021	USA	81.6 (70–97)	76 Older Adults 55 Females 21 Males	Qualitative study using phone interview	March 28–April 20, 2020
Clarissa Giebel 2022	Uganda		30 Older Adults 23 Females 7 Males	Qualitative study using semi-structured interviews	
Ilse Bloom 2022	UK	83.0 (81.5–85.8)	12 Older Adults 5 Females 7 Males	Qualitative study using serial telephone interviews	From March to October 2020
Saideh Garousi 2022	IRAN	… (65–85)	15 women over the age of 65	Qualitative phenomenological method using interviews	Between December 11, 2020, and March 5, 2021
A. R. Gonçalves 2021	Brazil, Italy, Portugal, United States	… (65.8–72.4)	25 Older Adults 19 Females 6 Males	Qualitative study with semi-structured interviews	From May to June 2020

The JBI checklist was used to assess the quality of the articles. The JBI tool for evaluating qualitative studies has ten items. According to the JBI checklist, the selected studies’ quality was optimal. Most of the selected articles were of superior quality, and most of the necessary items in qualitative research were addressed (Table 2). Most of the articles in Item 6 of the checklist, which was about locating the researcher culturally or theoretically, did not provide information [15, 18, 23, 26, 28, 31–33]. Also, most articles did not address the researcher’s influence on the research and vice versa (Table 2).

**Table 2:** Evaluation of the quality of articles with the JBI checklist

Items	Studies												
	Miriam Verhage 2021	Mahapatra 2021	Mikael 2021	Turner 2021	Alexandra 2021	Richard 2022	Rotenberg 2021	Jessica 2021	Heather 2021	Clarissa Giebel 2022	Ilse Bloom 2022	Saideh Garousi 2022	A. R. Gonçalves 2021
Item1	Y	Y	Y	Y	Y	Y	Y	Y	Y	Y	Y	Y	Y
Item2	Y	Y	Y	Y	Y	Y	Y	Y	Y	Y	Y	Y	Y
Item3	Y	Y	Y	Y	Y	Y	Y	Y	Y	Y	Y	Y	Y
Item4	Y	Y	Y	Y	Y	N	Y	Y	Y	Y	Y	Y	Y
Item5	Y	Y	Y	Y	Y	Y	Y	Y	Y	Y	Y	Y	Y
Item6	N	Y	Y	N	N	Y	N	N	N	N	Y	N	N
Item7	Y	N	N	N	N	N	Y	N	N	N	N	N	Y
Item8	Y	Y	N	Y	Y	Y	Y	N	Y	Y	Y	Y	Y
Item9	Y	Y	Y	Y	Y	Y	Y	Y	N	Y	Y	N	Y
Item10	Y	Y	Y	Y	Y	Y	Y	Y	Y	Y	Y	Y	Y
**JBI Score**	90%	90%	80%	80%	80%	90%	90%	70%	70%	80%	90%	70%	90%
**Quality of articles**	superior	superior	high quality	high quality	high quality	superior	superior	medium high	medium high	high quality	superior	medium high	superior

Y = Yes N = No

scoring: yes (2), no (0), and unclear (1)

70 -79% medium high quality

80–89% high quality

90% and above ranked as superior

Based on the extraction and combination of data, the challenges, and stressful experiences caused by the COVID-19 pandemic were classified into six themes: health management challenges, physical and social distancing challenges, economic challenges, media and bad news challenges, direct threats of the COVID-19 virus, and challenges related to the use of new communication technologies (Table 3).

**Table 3:** Challenges and stressful experiences caused by the COVID-19 pandemic

Health & Health management challenges	Closure of clinics and medical centers Doubts about the quality of face-to-face or virtual healthcare Changes in eating habits (like eating more snacks between meals) and more cravings for less healthy foods Weight Gain Decreased physical activity Increase in mental problems Sleep problems (decreased sleep at night and increased napping during the day)
Economic challenges	Disruption of work and income-generating activity Reduced job security Decrease in income Increased risk of fraud in online activities
Stress caused by quarantine	Experience feeling more alone and isolated Disruption of leisure time Disruption of daily structure Loss of social interaction (inability to participate in voluntary work, participation in groups, sports classes, religious activities, funerals, and shopping centers) Reduced energy and a lack of patience
Challenges of using communication technologies	Poor technical skills in using new communication technologies Fear of replacing virtual communication with real communication Fear of sharing personal information on social media platforms
Stress caused by media and bad news	Anxiety caused by the news of increasing deaths The presence of ageism in pandemic news broadcasting Increased death anxiety due to hearing pandemic news
Virus threats	Fear of death for oneself and loved ones due to infection The virus is out of control Fear of leaving home or going out Anxiety about getting infected with the virus Worry about the uncertainty of treatment Concerns about vaccine function

Older people used five main types of strategies to deal with the stress caused by the COVID-19 pandemic: protective strategies, avoidance strategies, maintaining social connections, meaning-based strategies, and fun strategies (Table 4).

**Table 4:** Older adults’ stress-coping strategies in response to the COVID-19 pandemic

Coping type	Examples of actions taken
Protective strategies	Actions to prevent the spread of virus: Social distancing, reduction of social interactions, and adherence to COVID-19 prevention protocols [18, 23–30, 33] Shopping during “senior hours”, reducing shopping trips or using home delivery services [27, 29, 30] Using the recommended personal protective equipment, such as goggles or a hat [26] Avoiding unnecessary purchases and minimizing the frequency of food purchases [26] Limiting outdoor meeting with family or friends [26, 30] Increased consumption of canned and frozen foods (strategic and creative meals) [23, 26] Using online counseling and health services [25] Participation in online religious groups [29] Actions to reduce sensitivity to the virus: Practicing health-promotion behaviors such as healthy eating, exercise, vaccination, and taking supplements to strengthen the immune system [23, 24, 26–29] Using virtual sports classes or sports videos [23, 29] Using herbal and home remedies to strengthen the immune system [26]
avoidance strategies	Reducing the use of news, filtering negative news related to COVID-19, and selective listening to news [18, 26–28] distraction [18, 26, 28] Avoiding some activities, such as riding a bicycle or cutting firewood to prevent the possibility of injuries and medical attention [26] Avoiding crowded areas and places where prevention guidelines are not followed [29] Eating out less frequently [29]
maintaining social connections	More intimacy with spouse and talking with family members [24] Maintaining high-quality relationships with family, friends, and neighbors [15, 28, 33] Taking online courses and adapting to new technologies to keep in touch with loved ones [15, 24, 32, 33] Sending emails, handwritten letters, and joining and using virtual social networks [29, 32]
meaning-based strategies	Belief in God, acceptance of fate, and faith in destiny [24] Practicing spiritual methods, keeping a positive attitude, and gaining perspective from past struggles [15, 29, 30] Appreciation for current assets [23, 26, 29, 32] Viewing quarantine as a temporary situation [18, 26] Finding happiness in small things [23, 25, 28, 32] Being optimistic about the future, hoping to return to normal life, and having plans for enjoyable activities in the future [25, 26, 33] Helping others, doing voluntary work, and feeling purposeful [23] Finding a new meaning or purpose in life [24]
fun strategies	Replacing creative activities at home with outdoor activities [27, 30, 32] Doing fun activities during the day (watching TV, reading books, gardening, listening to music, cleaning the house) [15, 23, 29, 32, 33] Practicing yoga and meditation at home [23, 26, 27] Joining online social networks [29, 32, 33]

## Discussion

In this study, 13 qualitative studies were systematically reviewed to identify the stress caused by the COVID-19 pandemic and the coping strategies of older adults. The study’s findings showed that older adults faced various challenges and stresses during the COVID-19 pandemic, which were classified into six groups. To cope with these stresses, older adults have used a wide range of coping strategies, which have been classified into five groups in this study: protective strategies, avoidance strategies, maintaining social connections, meaning-based strategies, and fun strategies.

Extended quarantine during a pandemic is considered stressful, and the expected outcomes include increased levels of depression and anxiety, increased social isolation, and increased feelings of loneliness [34]. According to the findings of Brenda et al., the most common stressors reported by older adults during the COVID-19 pandemic were quarantine and restrictions, concern for others, and loneliness [35]. In this study, we categorized the challenges caused by the pandemic into six groups: health management, quarantine, economics, media and bad news, direct virus threats, and communication technologies. According to Ellison et al.’s research, eight areas of social relations, activity limitations, psychological, health, financial, global environment, death, and care at home have been identified as difficult challenges caused by this pandemic. Social interaction limitations (42.4% of participants) and activity restrictions (30.9% of participants) have been the most common problems caused by the COVID-19 pandemic [36]. Radwan et al. also classified the major challenges affecting older adults during the COVID-19 outbreak into five categories: misinformation, health issues, limited access to nutritional needs, and violence against older adults [37].

Coping strategies are behavioral and cognitive strategies for dealing with stressful situations and crises. There are different classifications for coping strategies [38]. Coping strategies were classified by Robert Folkman and Susan Lazarus as either problem-oriented or emotion-oriented [39]. Depending on the situation and conditions, older adults can employ various strategies to mitigate the effects of a stressful situation. Verhage et al. found that older adults mainly used emotion-based coping strategies may be caused by the incomprehensible nature of the COVID-19 crisis [18]. Each of the coping strategies in place can be effective in maintaining and improving people’s mental health. Coping strategies are not just an interaction between the person and the situation but a dynamic process in which dimensions such as duration, timing, and order of stressful factors may play an important role [39].

Protective strategies are active efforts to manage and solve problems caused by a stressful factor [36]. Protective strategies can also be classified as problem-oriented strategies. The problem-oriented coping strategy includes the person’s constructive actions in stressful conditions and tries to remove or change the source of stress [39]. According to studies, problem-oriented strategies improve psychological well-being and quality of life [40]. With COVID-19 as a stressful factor, preventive strategies include hand washing, mask-wearing, and social distancing that people can use directly and actively to prevent the virus’s spread and protect themselves [16]. Most studies showed that older people used these strategies to reduce their virus exposure and susceptibility. According to surveys, most older adults know the COVID-19 preventative protocols. They adopt protective strategies and preventive measures, including using masks and hand disinfectants and changing clothes when entering the house [33, 41].

Stress in the context of the COVID-19 pandemic also affects physical health. Staying healthy and maintaining physical mobility during the COVID-19 outbreak and implementing quarantine and social isolation are necessary for older persons [42]. Physical activity at home improves the immune system’s response and reduces susceptibility to the virus. The findings of this study demonstrated that exercising at home was one of the strategies adopted by older people to cope with a reduced level of physical activity. The most popular strategies utilized by older people in quarantine to enhance physical activity were backyard gardening projects [26, 28, 29], walking [23, 26, 29, 32], and using virtual sports classes or watching sports videos [23, 29].

When individuals experience unpredictable conditions, such as natural disasters, meaning-focused coping can help them adjust [43]. This strategy attempted to improve an individual’s evaluation of a situation and make beliefs, goals, and stressful events more consistent so that individuals can cope with stressful conditions [44]. Meaning-oriented strategies include attitudes and behaviors that help people positively reinterpret hardship, preserve hope, appreciate life, and engage in meaningful activities [45]. This strategy aimed to find a positive meaning in a negative experience. Coping strategies grounded in meaning are generally based on older adults’ lifetime experiences [46]. This group of coping strategies is sometimes classified as emotion-oriented [18]. In this study, a wide range of coping strategies used by older adults were classified as meaning-oriented. Gratitude [23, 26, 29, 32], finding joy in small things [23, 25, 28, 32], being optimistic about the future and hoping to return to normal life, and planning for enjoyable activities in the future were among the most popular strategies used by older adults (see Table 4). Nikolett et al. found that meaning-based coping is substantially associated with the reduction of stress, anxiety, and depression symptoms during the COVID-19 epidemic [45].

To avoid contracting COVID-19, older adults stayed at home and decreased their face-to-face social participation. However, many older adults reported continuing social communication virtually, such as through phone calls. In this study, the challenges of using communication technologies were categorized as one of the important challenges of the COVID-19 pandemic era. Technological issues such as poor or incompatible Internet connections, sensory problems (such as poor hearing), and discomfort caused by long-time use of computers (such as headaches and visual discomfort) were among the problems and challenges facing older adults when using communication technologies [26]. According to participants in the Greenwood-Hickman study, while virtual media helps individuals stay connected with friends and family, it is not always as satisfying as face-to-face engagement. However, virtual communication was easier for some people than face-to-face meetings [29]. In the study by Fiocco et al., despite some initial resistance and challenges experienced by some participants, older adults gradually realized the necessity of technology to connect with their social network during the pandemic and also pointed out the benefits of this technology in work affairs, online worship, and online learning. Regarding these technologies, some participants acknowledged that learning to use some communication tools may be difficult for them. The government should provide social assistance to support the use of technology for this class of society [28]. Communication technologies can significantly help older adults and their caregivers during pandemics. The “*digital divide*” and limited access to technologies among older adults deserve consideration to reduce such problems in future pandemics [47].

The news on disease-related deaths is one of the main challenges facing older adults during the COVID-19 pandemic. COVID-19 was described as an age-related condition that caused worry in older people. Although many older adults used the media to learn about the spread of the coronavirus and recommended safety measures at the beginning of the outbreak, they reported the negative impacts of disease-related death news on their mental health [28]. Losada-Baltar et al. found that older adults, who are more exposed to news on COVID-19, experience higher psychological distress [48]. The results of our review showed that older adults have used different strategies to cope with this issue. Reducing the time spent listening to the news, filtering negative news on COVID-19, and selectively listening to the news has been the most important strategies for older adults to cope with this situation. (See also Table 3)

Recreation and hobbies are the main components of a healthy life for older adults [49]. With the spread of COVID-19, most recreational facilities for older adults, such as indoor sports facilities, parks, and cultural places, were closed. Most group activities, such as voluntary activities and clubs, were also implemented with severe restrictions [50]. Our study showed that older adults entertain themselves in different ways, such as watching TV, reading books, gardening, listening to music, cleaning the house, and joining online social networks (see Table 4). Kim et al.‘s study showed that during the COVID-19 pandemic, the participation rate of older Korean adults in entertainment and sports activities has increased. In contrast, their participation rate in cultural, tourism, and social activities has decreased. Studies showed that the proportion of older adults who spend their leisure time on active and social activities has decreased, and the ratio of older adults who spend their leisure time on passive activities has increased [50].

Most studies collect data through telephone interviews (see Table 1). Although telephone interview can deprive the researcher of establishing non-verbal communication with respondents [51], it is the best method of collecting information in situations such as pandemics and infectious diseases, which can ensure the safety of people by preventing the transmission of diseases. Telephone interviews also increase the feeling of anonymity in some sensitive subjects, calm them down, and reduce the effects of the interviewer’s presence [52].

### Limitations

In this review, studies of older adults living in different geographic regions were analyzed, including Europe [18, 25, 30], Asia [24, 32], the United States [15, 23, 27, 29], and Africa [31]. The findings should be interpreted in light of the studies’ diverse sociocultural contexts. Future studies could examine participants exclusively from similar geographic regions to avoid issues of geographic heterogeneity. Knowing about the coping strategies of older adults during the pandemic can be useful for activists in the field of older adults’ health. The findings of this research outlined the stress experienced by older adults during the COVID-19 pandemic, as well as their coping strategies, which may be used in the formation of policies on elderly health promotion in the post-COVID era.

## Conclusion

Older adults are experiencing a lot of stress and challenges during the COVID-19 pandemic. Depending on the situation and conditions, older adults can use a variety of strategies to reduce the effects of the stressful situation of COVID-19. In most cases, older adults deal with these challenges using simple strategies based on previous experiences and learnings, that do not necessitate a large budget or many resources. Training in effective coping strategies in middle age can prepare them for old age and guarantee their psychological security to some extent. In some cases, such as the use of communication technologies, older adults require educational interventions. The supervision and cooperation of health professionals in producing media content during the outbreak of the pandemic can effectively reduce stress on older adults and prevent problems such as ageism. Experts in geriatrics can use this research’s findings in designing their clinical interventions to familiarize middle-aged and older adults with efficient coping strategies and strengthen social support in the post-COVID era.

### Electronic supplementary material

Below is the link to the electronic supplementary material.

Supplementary Material 1

## Data Availability

All data has been summarized and provided in the supplementary files. Please contact the corresponding author for data requests.
